# Supplementary figures and images for: Effect of salt stress and nitrogen supply on seed germination and early seedling growth of three coastal halophytes
Source: PeerJ. 2022 Oct 7;10:e14164. doi: 10.7717/peerj.14164 (PMC9549898; doi:10.7717/peerj.14164)

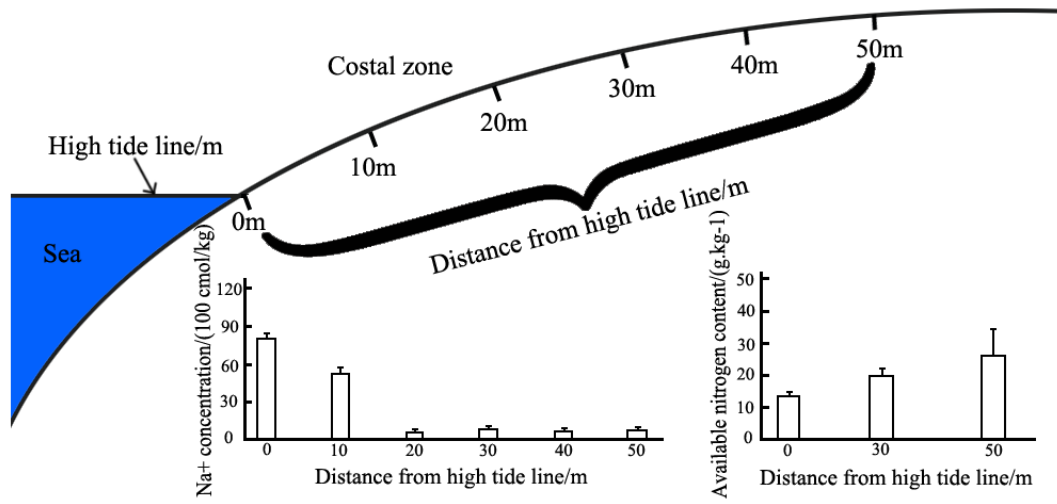

Supplement: Supplemental Information 1 [file peerj-10-14164-s001.pdf]

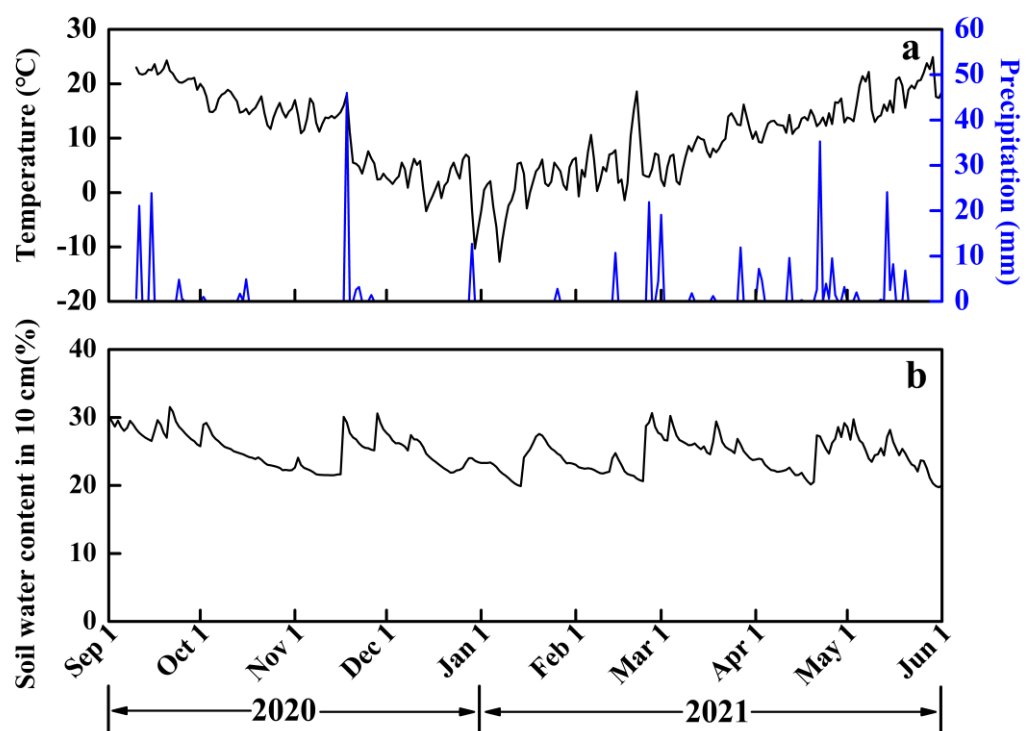

Supplement: Supplemental Information 2 [file peerj-10-14164-s002.pdf]
